# Effects of protein transduction domain (PTD) selection and position for improved intracellular delivery of PTD-Hsp27 fusion protein formulations

**DOI:** 10.1007/s12272-016-0786-9

**Published:** 2016-07-05

**Authors:** Qurrat Ul Ain, Jong Hwan Lee, Young Sun Woo, Yong-Hee Kim

**Affiliations:** 1 Department of Bioengineering, Institute for Bioengineering and Biopharmaceutical Research, Hanyang University, 17 Haengdang-dong, Seongdong-gu, Seoul, 04763 Korea; 2 BK 21 Plus Future Biopharmaceutical Human Resources Training and Research Team, Department of Bioengineering, Institute for Bioengineering and Biopharmaceutical Research, Hanyang University, 17 Haengdang-dong, Seongdong-gu, Seoul, 04763 Korea

**Keywords:** Fusion proteins, Selection and position of protein transduction domains (PTDs), Heat shock protein 27, Hypoxia

## Abstract

Protein drugs have attracted considerable attention as therapeutic agents due to their diversity and biocompatibility. However, hydrophilic proteins possess difficulty in penetrating lipophilic cell membrane. Although protein transduction domains (PTDs) have shown effectiveness in protein delivery, the importance of selection and position of PTDs in recombinant protein vector constructs has not been investigated. This study intends to investigate the significance of PTD selection and position for therapeutic protein delivery. Heat shock protein 27 (Hsp27) would be a therapeutic protein for the treatment of ischemic heart diseases, but itself is insufficient to prevent systemic degradation and overcoming biochemical barriers during cellular transport. Among all PTD-Hsp27 fusion proteins we cloned, Tat-Hsp27 fusion protein showed the highest efficacy. Nona-arginine (9R) conjugation to the N-terminal of Hsp27 (Hsp27-T) showed higher efficacy than C-terminal. To test the synergistic effect of two PTDs, Tat was inserted to the N-terminal of Hsp27-9R. Tat-Hsp27-9R exhibited enhanced transduction efficiency and significant improvement against oxidative stress and apoptosis. PTD-Hsp27 fusion proteins have strong potential to be developed as therapeutic proteins for the treatment of ischemic heart diseases and selection and position of PTDs for improved efficacy of PTD-fusion proteins need to be optimized considering protein’s nature, transduction efficiency and stability.

## Introduction

Since 2001, more than two hundred new FDA approvals of protein drugs and peptides have emerged in mainstream therapeutics. As a substitute for small molecules and gene therapy, they represent a significant percentage of the biopharmaceutical market (Arthanari et al. 2010; Walsh 2010). Protein drugs show highly diverse structures and wide biological efficacies and thus have a considerable role in both therapeutics and bio imaging. Protein drugs are safer and more biocompatible than therapeutic gene delivery (Tan et al. 2010). Hydrophilicity and large molecular weight of protein drugs are core limiting factors to their cellular level delivery (Carter 2011). Selective permeability and lipophilic property of plasma membrane prohibits protein drug delivery into cells. Additionally, protein structure should not be broken for preventing their dysfunction (Wang 2005). Peptide and protein drugs are also prone to proteases and unspecific proteolysis. Hence, efficient protein delivery through cell membrane could be an arduous challenge.

Protein transduction domains (PTDs) conjointly known as cell-penetrating peptides (CPPs) are a class of diverse peptides, typically with 5–30 amino acids. In contrast to many proteins, they can target intracellular proteins. Importantly, PTDs can also incorporate other biomolecules such as proteins, DNA, antibodies and contrast agents into cells, thus offering great potential as future therapeutics. Among several alternative carrier-mediated delivery systems, use of CPPs as a carrier for cellular delivery is taken into account as one of the best strategy (Snyder and Dowdy 2004; Deshayes et al. 2005; Gupta et al. 2005). PTDs can also efficiently transport cargos which are several times greater in molecular weights than their own and are efficient for a large range of cell types (Van den Berg and Dowdy 2011).

Trans-activator of transcription (Tat: GRKKRRQRRRPQ), first discovered PTD, and poly-arginine represents the cationic class of PTDs. HIV-1 Tat has showed effective transduction capability for various cargos (Lim et al. 2010; Won et al. 2010; Arthanari et al. 2010; Zhang et al. 2012). It consists of positively charged transduction through negatively charged phospholipid on cell membrane. Since then, positively charged amino acids are highlighted as a key factor for transduction capacity. Studies on poly-arginine (from R3 to R12) have evidenced that octa-arginine (R8) is minimal sequence for cellular uptake, and by increasing number of arginine, cellular uptake can be increased. Later it was shown that octa-arginine (8R) and nona-arginine (9R) showed more transduction as compared to undeca-arginine (11R) and dodeca-arginine (12R). Studies suggest that for efficient cellular internalization at least eight positive charges of cationic PTDs are needed. Among cationic PTDs, charged residues have a more crucial role in cellular uptake.

Even after intense research on PTDs, the exact mechanism of PTDs internalization into cytoplasm of these PTDs remains under intense investigation and is not fully understood. Energy-independent pathways, direct transduction by hydrophobic interaction between cell membrane and hydrophobic amino acids of Tat (Ziegler 2008; Schmidt et al. 2010; Mishra et al. 2011) and endocytic pathway are suggestions still open to debate. It seems clear that out of all, two types of mechanisms coexist, which dissent dramatically in terms of efficacy. In addition to PTDs direct transduction through electrostatic interactions on lipid bilayer (Herbig et al. 2005), an energy-dependent primary endocytic pathway is responsible for intracellular transport (Wadia et al. 2004). Whereas a Tat peptide shows various translocation mechanisms according to their cargo, 9R mainly transports a cargo by endocytosis mechanism (Takeuchi et al. 2006). It is now evident that various PTDs and PTDs–cargos can enter cells by different mechanisms (Duchardt et al. 2007), therefore, significance of PTD selection and position should be emphasized and considered an important factor for effective penetration of protein.

**Fig. 1:**
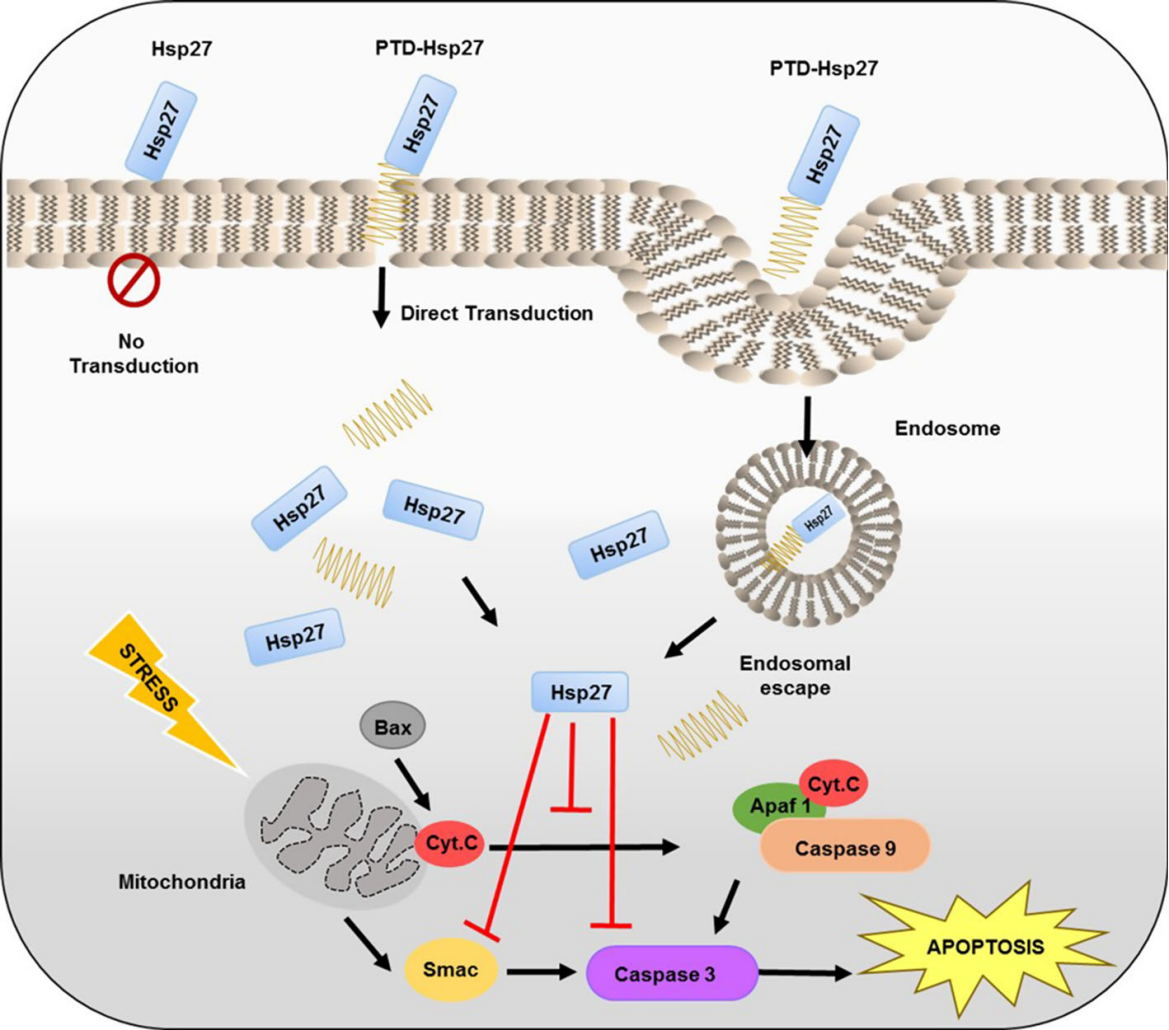
Schematic illustration of transduction of PTD-Hsp27 fusion proteins and their therapeutic effects on apoptosis

In this study, we intend to prove significance of PTD selection by using PTD-conjugated heat shock protein 27 (Hsp27) fusion proteins. Hsp27 is a chaperone with a molecular weight of 27 kDa. It inhibits apoptosis against hypoxia in cells by interacting directly with caspase activation components (Tan et al. 2009), especially when linked with PTDs, it showed significant effects against hypoxia as shown in Fig. 1 (Kwon et al. 2007; Liu et al. 2014). 9R was conjugated to C-terminus and N-terminus of Hsp27-protein to compare effect of PTDs by their locations. Tat conjugated to Hsp27 was used as a positive control (Tan et al. 2009). As 9R-Hsp27 showed better results than Hsp27-9R, Tat was conjugated at N-terminal of Hsp27-9R to observe improvement in Hsp27-9R. After anti-apoptotic efficacy comparison, it was concluded that N-terminal conjugated PTD-Hsp27 fusion proteins have more protective effects than C-terminal.

## Materials and methods

### Materials

H9c2 cells (Rat cardio myoblast) were obtained from a Korean cell line bank (Seoul, Korea). High glucose Dulbecco’s Modified Eagle’s Medium (DMEM) and fetal bovine serum (FBS) were purchased from WelGENE (Seoul, Korea). Cy5-Intracellular nucleic acid localization kit was obtained from Mirus Bio Corporation (Madison, USA). DC protein assay kit and Bovine Serum Albumin (BSA) standard were purchased from Bio-Rad Laboratories (Hercules, CA). Dialysis Membrane (MWCO: 12-14,000) was obtained from Spectrum Laboratories (Richmond, CA).

### Cloning

To construct PTD-Hsp27 fusion proteins, Hsp27 a cDNA sequence from PAcGFP1-N, obtained from Invitrogen (La Jolla, CA), was inserted into pET28a bacterial expression vector. First, 9R (CGA CGT CGC CGG CGT CGA CGT CGA CGG) or Tat (p47–57; TAT GGC AGG AAG AAG CGG AGA CAG CGA CGA CGA) peptide were cloned into pET28a vector to construct pET28a-9R or pET28a-Tat vectors with poly-histidine (HHHHHH) affinity tag at C- terminal. Then, to clone Hsp27 into pET28a-9R or pET28a-Tat vectors, Hsp27 cDNA was amplified and cloned into pET28a-9R and pET28a-Tat vectors between each restriction enzyme sites. Finally, pET28a-T-Hsp27, pET28a-9R-Hsp27, pET28a-Hsp27-9R, and pET28a-T-Hsp27-9R vectors were verified by DNA sequencing. All plasmid constructs for PTD-conjugated proteins were synthesized and analyzed by Cosmo Genetech (Seoul, Korea).

### Expression and purification of plasmid constructs for PTD-conjugated Hsp27 fusion proteins

Plasmid constructs for PTD-conjugated proteins were transformed to *Escherichia coli* BL21 strain, obtained from Strata gene (La Jolla, CA). Bacteria were cultured in LB medium with 50 μg/ml kanamycin at 37 °C with shaking for 4 h until the OD600 value reached 0.6–0.8. Protein expression was stimulated by adding 1 mM isopropyl β-d-1-thiogalactopyranoside and shaking for 16 h at 26 °C. Cell pellets were collected by centrifugation at 4,800 × *g*-force for 10 min and cells were re-suspended by using lysis buffer with 100 mM phenyl-methyl-sulfonyl-fluoride. After re-suspension, cells were sonicated for 25 s each time and then cell debris was separated from proteins by centrifugation at 27,500 × *g*-force for 25 min. Separated proteins were then filtered through 0.45 μm filter. With elution buffer, proteins were purified by immobilized metal affinity chromatography with FPLC using Ni–NTA resin column. Purified proteins were solubilized in elution buffer, and impurities were removed by using dialysis membrane (MWCO: 12-14,000), in phosphate buffered saline (PBS) buffer. Purified fusion proteins were aliquoted respectively and stored at 4 or −70 °C.

### Immunoblotting

PTD-conjugated fusion proteins were identified by immunoblotting. After electrophoresis on 15 % SDS-polyacrylamide gel, fusion proteins were transferred to PVDF membrane overnight at 4 °C following blockage with 5 % skim milk. Transferred proteins were then treated with primary His-Tag polyclonal antibody, purchased from Cell Signaling (Danvers, MA), over-night at 4 °C followed by treatment with secondary anti-rabbit HRP-linked IgG for 2 h at 4 °C. Membranes were visualized with ECL Plus solution.

### Cell culture

H9c2 cells were cultured in DMEM (d-glucose: 4,500 mg/l, l-glutamine, sodium pyruvate: 110 mg/l, sodium bicarbonate) high glucose containing penicillin (100 U/ml) streptomycin (100 mg/ml) and 10 % FBS. Cells were maintained at 37 °C with 5 % CO_2_.

### Cytotoxicity assay

MTT assay was performed to measure cytotoxicity of PTD-conjugated fusion proteins. H9c2 cells were seeded in 96 well plate for 24 h at 37 °C. Cells were then treated with 1–10 μM PTD-Hsp27 fusion proteins for 4 h. To check cell viability, MTT reagent was added to cells following incubation at 37 °C for 2 h. The absorbance was measured by an ELISA plate reader at 570 nm. Cytotoxicity assay was repeated three times with four replicates.

### Immunofluorescence assay

To observe intracellular uptake, H9c2 cells were seeded onto cover glass for 24 h. PTD-conjugated fusion proteins were labeled with cy5 as described by manufacturer. Cells were treated with PTD-conjugated fusion proteins with 5 μM PTD-Hsp27 concentration for 24 h. Cells were then washed with ice-cold PBS and counterstained with DAPI. To detect intracellular transduction, cells were immediately fixed at 4 °C with 3.7 % formaldehyde. Transduction of PTD-conjugated fusion proteins was observed by a confocal microscope (Carl Zeiss; Jena GmbH, Germany).

### Flow cytometry analysis

Flow cytometry analysis was performed to confirm PTD-Hsp27 fusion protein’s transfection. H9c2 cells were seeded in six well plates for 24 h. PTD-Hsp27 fusion proteins were labeled with alexa488 according to manufacturer instructions. 5 μM of labelled fusion proteins were added and incubated for 24 h. Cells were collected by trypsinization and centrifugation. Single cell suspension was prepared in FACS buffer (2 % FBS, 0.02 % sodium azide/PBS). Internalization of PTD-Hsp27 fusion proteins was evaluated by a BD FACS Caliber flow cytometer (San Jose, CA).

### Hypoxia induction

In order to test anti-apoptotic effects of PTD-Hsp27 fusion proteins, H9c2 cells were exposed to apoptotic condition by treatment with sodium arsenide, NaAsO_2_. The 50 % inhibitory concentration of cell viability (IC50) was screened at NaAsO_2_ concentrations below 15 μM. Cells were incubated for 24 h at 37 °C under 95 % N_2_, and 5 % CO_2_ in a hypoxia chamber. Cell viability was measured by MTT assay.

### Apoptosis assay

H9c2 cells were seeded in 96 well plates and treated with 10 μM NaAsO_2_ and 5 μM of Hsp27 and PTD-conjugated Hsp27 fusion proteins. Anti-apoptotic effects of PTD-Hsp27 fusion proteins were measured by MTT assay following 24 h incubation with proteins.

### Statistical analysis

All data are represented as mean ± SD (standard deviation) (n = 3). Statistical analysis was performed with Student’s *t* test. P values less than 0.05 were considered statistically significant.

## Results

### Expression and purification of PTD-conjugated Hsp27 fusion proteins

**Fig. 2:**
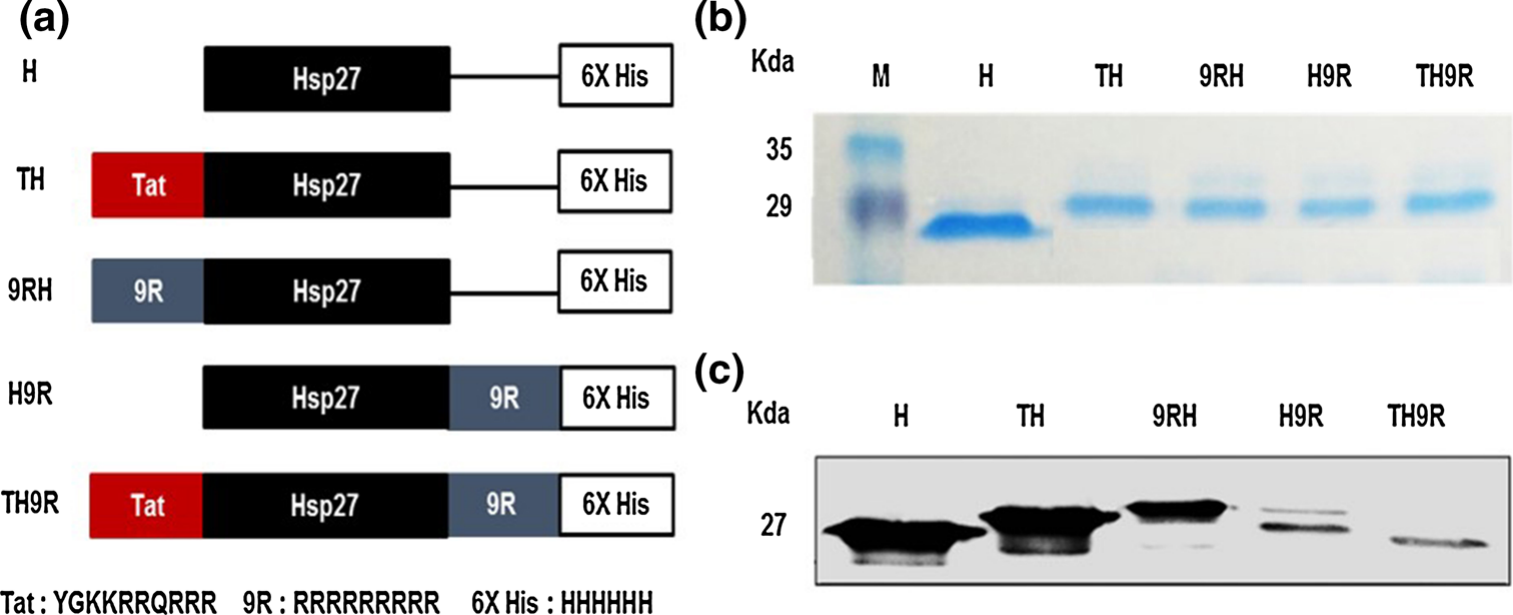
PTD-Hsp27 fusion proteins purification and expression. **a** Schematic structures of vector constructs **b** identification of PTD-Hsp27 fusion proteins by SDS-PAGE and **c** western blot. *H* Heat shock protein 27, *TH* Tat-Hsp27, *9RH* Nona-arginine-Hsp27, *H9R* Hsp27-Nona-arginine, *T-H9R* Tat-Hsp27-Nona-arginie

The pET28a vector was used to construct pET28a-Tat and pET28a-9R vectors with C-terminal poly histidine (6× His) affinity tag. Hsp27 genes were cloned into pET28a-Tat and pET28a-9R vectors. Vector designs of PTD-Hsp27 fusion proteins and their nucleotide sequences were confirmed by sequence analysis. Tat and 9R were cloned to C-terminus or N-terminus of Hsp27. Vector constructs of Hsp27 recombinant proteins are shown in Fig. 2a.

PTD-conjugated fusion proteins were collected from BL21 *E. coli* lysates and purified by nickel affinity chromatography using a Ni–NTA resin column with FPLC. Ni–NTA resin column in FPLC was used to capture and purify 6×  His tag-labeled proteins. SDS-PAGE was used to identify purified PTD-Hsp27 fusion proteins as shown in Fig. 2b. After electrophoresis on 15 % SDS-polyacrylamide gel, fusion proteins were transferred to PVDF membrane overnight at 4 °C and then treated with His-tag polyclonal antibody and secondary anti-rabbit HRP-linked IgG. Western blot results for PTD-Hsp27 are shown in Fig. 2c.

### In vitro cytotoxicity assay

**Fig. 3:**
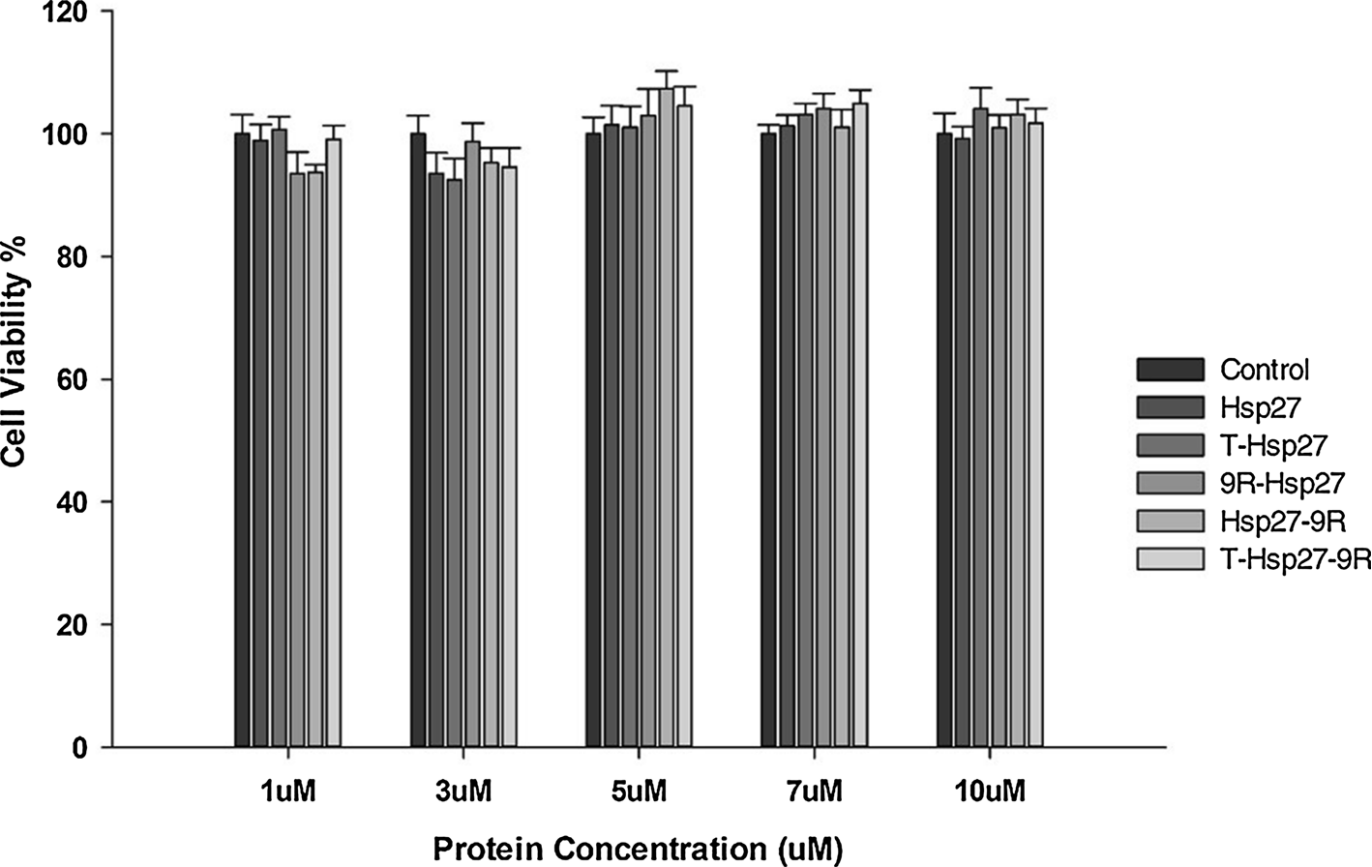
In vitro cytotoxicity of PTD-Hsp27 fusion proteins in H9c2 cells

The cytotoxicity of all PTD-Hsp27 recombinant fusion proteins in H9c2 cells was tested in dose dependent mode from 1 to 10 μM. Cells were treated with 1, 3, 5, 7 and 10 µM PTD-Hsp27 purified fusion proteins for 24 h. MTT assay was used to determine cell viability. As shown in Fig. 3, no significant cytotoxicity was observed in any of the PTD-Hsp27 fusion proteins treated groups within the assigned dose-range of 1–10 μM. For further experiments, the protein concentration was fixed at 5 μM. These results suggest that PTDs such as 9R and Tat could be used as safe therapeutic protein carriers with minimum toxicity in mammalian cells regardless from their site of attachment with therapeutic protein Hsp27.

### Intracellular transduction of PTD-Hsp27 fusion proteins

To function against hypoxia, Hsp27 should be located in the cytosol. However, it is difficult to penetrate cell membranes for Hsp27 itself due to its hydrophilic and macromolecular characteristics. The recombinant fusions of PTD with Hsp27 were considered to enrich the transduction efficiency of Hsp27. Although transduction mechanisms of PTD fusion proteins are still controversial, the high transduction efficiency of T- and 9R-Hsp27 fusion proteins was expected. In order to visualize internalization and retention of PTD-Hsp27 fusion proteins in H9c2 cells, confocal laser scanning microscopy (CLSM)was used and intracellular trafficking of proteins was observed after 24 h of treatment.

**Fig. 4:**
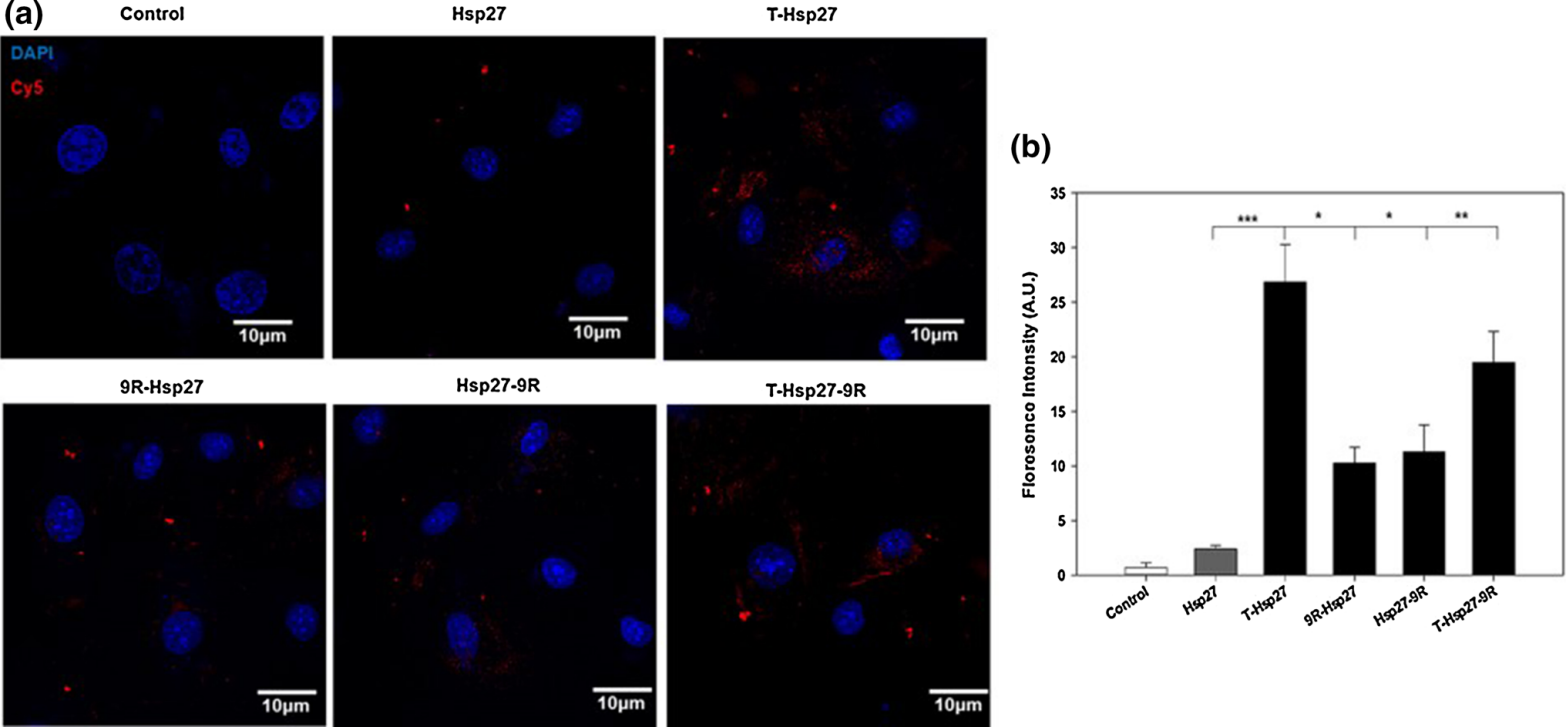
Intracellular uptake of PTD-Hsp27 fusion proteins. Cells were incubated with cy5 conjugated PTD-Hsp27 fusion proteins for 24 h. CLSM images were merged with PTD-conjugated fusion proteins following counterstaining with DAPI

PTD-Hsp27 fusion proteins were conjugated by Cy5, following counter-staining with DAPI. As shown in Fig. 4a, florescence of Cy5 in T-Hsp27 group was dispersed more widely in cytoplasm than other groups (H, 9RH, H9R, TH9R), in H9c2 cells. In case of 9R-Hsp27, Hsp27-9R and T-Hsp27-9R groups, less Cy5 florescence intensities were detected in cytoplasm. Among 9R-Hsp27 and Hsp27-9R fusion proteins, 9R-Hsp27 showed less florescence. Florescence intensities were quantified by Image J software.

### Flow cytometry analysis of PTD-Hsp27 fusion proteins

**Fig. 5:**
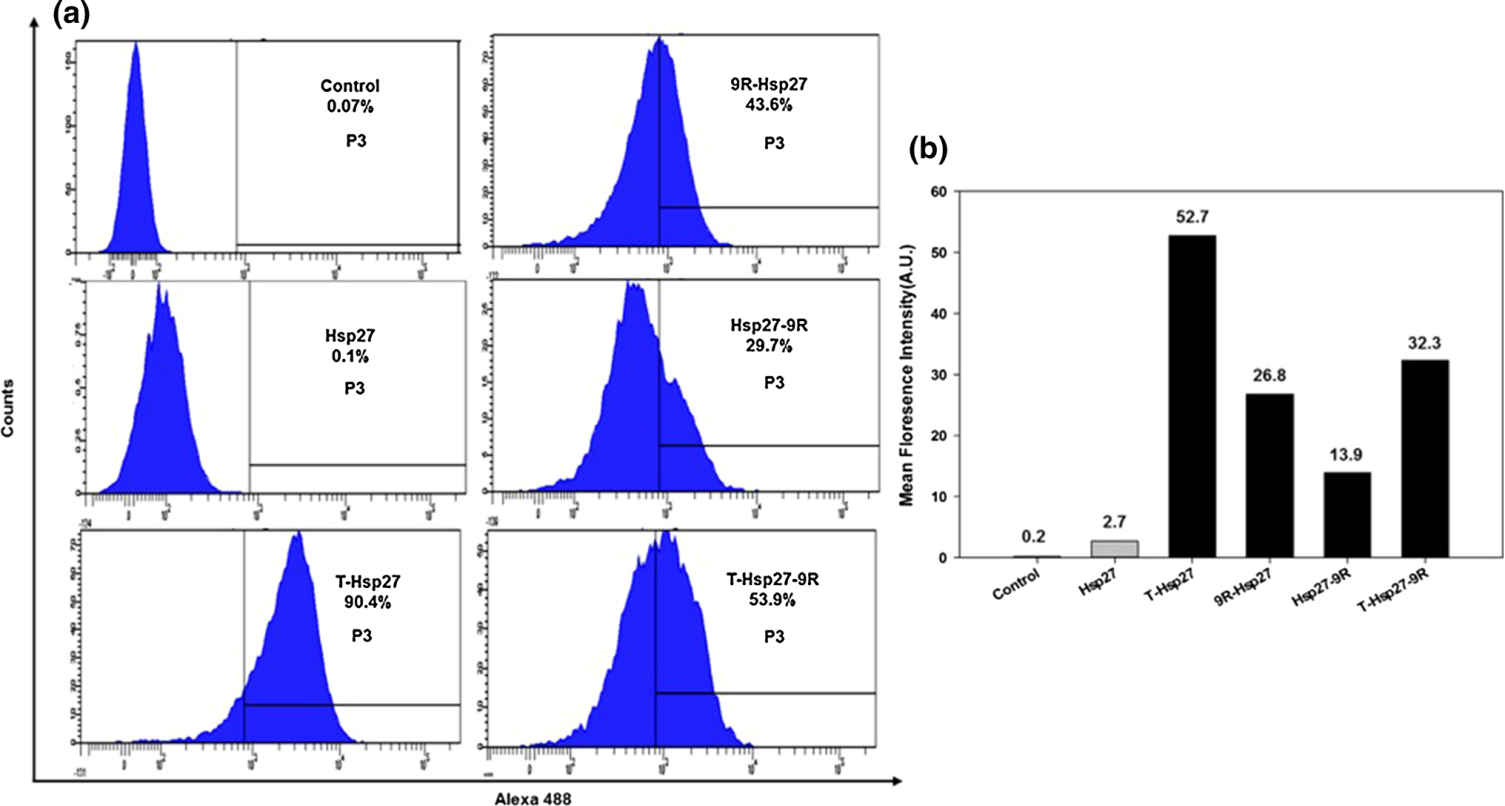
Flow cytometry analysis of PTD-Hsp27 fusion proteins. H9c2 cells were transfected with alexa488 labelled Hsp27 and PTD-Hsp27 fusion proteins, (T-Hsp27, 9R-Hsp27, Hsp27-9R and T-Hsp27-9R). The fluorescence intensity of Alexa488 was measured after 24 h by FACS analysis

For further comparison of PTD-Hsp27 fusion protein’s transfection efficiency, flow cytometry was performed and mean fluorescence intensity (MFI) from triplicate for each group was recorded. PTD-Hsp27 fusion proteins were labeled with alexa488 according to manufacturer instructions and excessive dye was removed by dialysis in PBS. Cells were treated with PTD-Hsp27 fusion proteins at 5 μM concentration and incubated at 37 °C for 24 h. After incubation, cells were collected by trypsinization followed by washing and centrifugation. Single cell suspension was prepared in FACS buffer (2 % FBS, 0.02 % sodium azide/PBS). Internalization of PTD-Hsp27 fusion proteins was evaluated by a FACS Canto II Caliber flow cytometer (BD Franklin Lakes, NJ) and data was analyzed by Cell Quest Pro software. Figure 5a represents that T-Hsp27 fusion protein retains 90.4 % transfection efficiency significantly higher than 9R-Hsp27 (43.6 %), Hsp27-9R (29.7 %) and T-Hsp27-9R (53.9 %). This data confirms that Tat provides better transfection efficiency for Hsp27 than 9R. As shown in Fig. 5b), the MFI values were also measured with transfection of Alexa 488-conjugated PTD-Hsp27 fusion proteins. The mean fluorescence intensity of T-Hsp27 was 52.7 and relative values of 9R-Hsp27, Hsp27-9R and T-Hsp27-9R groups were about 26.8, 13.9 and 32.3, respectively.

### Anti-apoptotic effect of PTD-Hsp27 fusion proteins in hypoxia condition

Anti-apoptotic effects of PTD-Hsp27 fusion proteins in hypoxic conditions were studied to determine the impact of Hsp27 as protective agent. Anti-apoptotic effects of PTD-Hsp27 were observed by measuring cell viability of H9c2 cells in hypoxic conditions.

Sodium arsenite (NaAsO_2_), an appropriate chemical stressor through the generation of reactive oxygen species (ROS), (Watson et al. 1996) was used to generate hypoxic state of H9c2 cells. Studies also show that NaAsO_2_ can induce a dose-dependent increase of ROS in cultured human cells. Therefore to optimize IC50, MTT assay was performed with various concentration of NaAsO_2_. At 10 μM NaAsO_2_ concentration, 51.3 % cell viability was observed, hence, 10 μM NaAsO_2_ was used in further experiments to generate hypoxic environment.

**Fig. 6:**
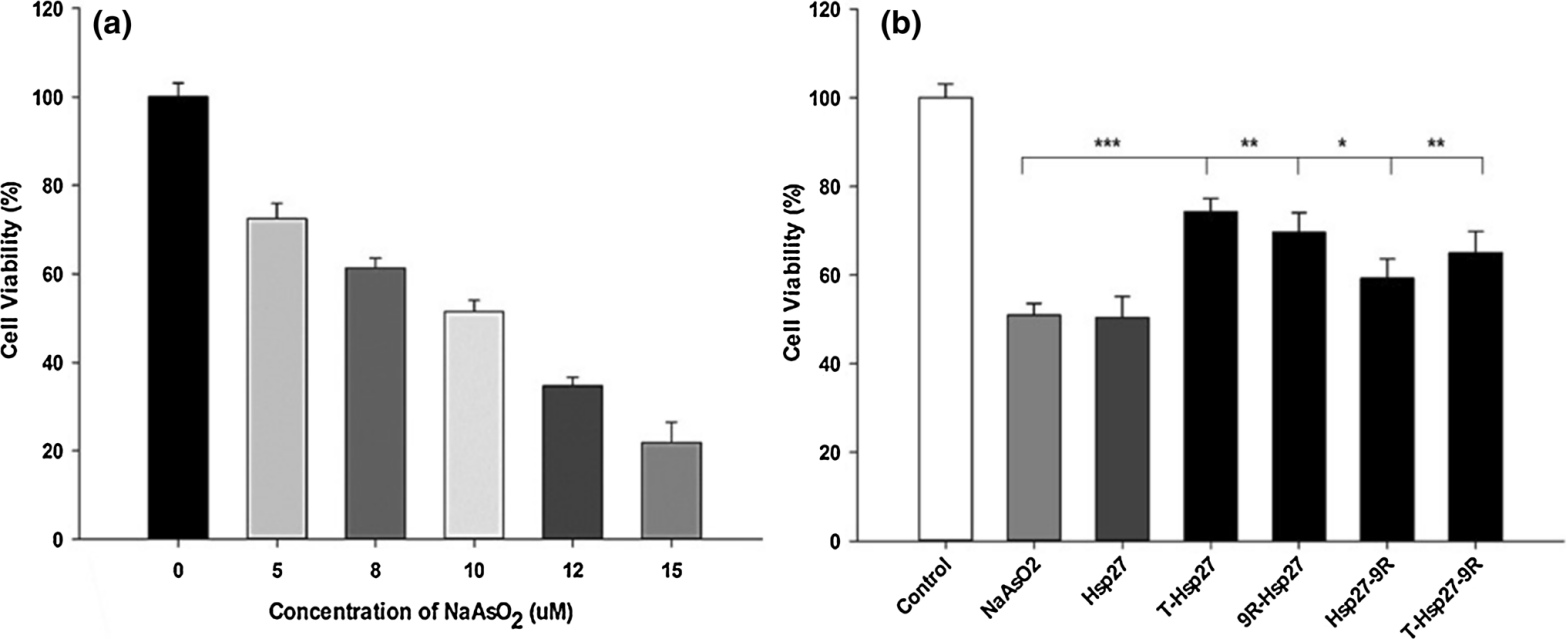
In vitro anti-apoptotic effects of PTD-conjugated fusion proteins. **a** IC50 was measured with different conc. of NaAsO_2_. **b** Cell viability was measured to check anti-apoptotic activity by 5 µM PTD-Hsp27

To evaluate the effects of PTD-Hspp27 fusion proteins in hypoxic condition, H9c2 cells were treated with 5 μM of PTD-Hsp27 fusion proteins for 24 h. Cell viability in T-Hsp27 treated cells increased about ~25 % while there was no significant effect in Hsp27 treated cells. Cell viability in 9R-Hsp27, Hsp27-9R and T-Hsp27-9R groups increased about ~20, 17 and 15 %, respectively (Fig. 6b). In the case of 9R-Hsp27, Hsp27-9R and T-Hsp27-9R, the protective effects were much higher than with just Hsp27, indicating that Hsp27 itself could not be transduced efficiently into cells. These results indicate that transduction of Hsp27 into cells is essential for the protection of cells against hypoxia and that Hsp27 is an anti-hypoxic protein.

## Discussion

Most intractable in vivo barriers for protein drug candidates are the immunological responses caused by the recognition of the protein as a foreign antigen. Key factors causing immunogenicity are impurities and aggregations, further resulting in loss of protein efficacy. To cope up with the immunogenicity issue, protein drugs need to be soluble and non-aggregated. Although the fusion protein drugs in market are generally safe and effective, the expensive cost of drugs has become another issue for chronic patients. In order to scale back the price and side effects, the drug candidate should be potent to keep the dose minimum. Furthermore, specific targeting of protein drug into disease sites should be achieved to minimize side effects and maximize efficacy.

PTDs are widely-tested carriers for DNA, RNA and proteins intracellular delivery (Del Gaizo and Payne 2003; Kaplan et al. 2005; Shokolenko et al. 2005). Here we prepared PTD-Hsp27 fusion proteins by cloning and fusing Hsp27 plasmid with PTDs to inhibit apoptosis in hypoxic conditions. Hsp27, a heat-shock protein, has crucial roles in many cellular processes including apoptosis, cellular differentiation and cytoskeleton dynamics. With the assistance of protein transduction domains, all of PTD-Hsp27 recombinant fusion proteins efficiently penetrated into cardiomyocytes. Amphipathic property and charge distribution are considered to be two important factors for cellular transduction of PTDs as the cellular transduction mechanism of and cationic property of PTDs alone is not absolute factor determining transduction efficiency of PTD because 9R containing more cationic amino acids without hydrophobic amino acids than Tat demonstrated less effective delivery of Hsp27 into cells. Taken together it can be observed that 9R conjugated with N-terminal of Hsp27 and T-Hsp27-9R can show more therapeutic efficacy than 9R conjugated to C-terminal of Hsp27 as they less aggregated and are more susceptible to produce therapeutic effects. Both MFI and florescence percentage values conclude that N-terminal conjugation improve transduction of PTD-Hsp27 fusion proteins and Hsp27 itself doesn’t have enough transduction efficiency to produce therapeutic effects. These results also endorse the previous results that N-terminal conjugated PTD-Hsp27, protected more cells against apoptosis than C-terminal conjugated PTD-HSp27, by interfering with apoptosis extrinsic pathway. T-Hsp27 showed the best transduction efficiency of all recombinant Hsp27 fused proteins with 9R and Tat combinations tested. N-terminal was proven to be important domain for the transduction of Hsp27 as Hsp27 conjugated with PTD at N-terminal showed better efficiency than Hsp27 conjugated with PTD at C-terminal. Moreover, cationic property of PTD alone is not a single parameter determining transduction efficiency of PTD followed by therapeutic efficacy as 9R showed less effective delivery of Hsp27 into cells than Tat even though it has more cationic amino acids than Tat.

The selection and position of PTDs for improved efficacy of PTD fusion proteins need to be optimized considering protein’s nature, transduction efficiency, stability and efficacy. PTD-Hsp27 fusion proteins, particularly T-Hsp27 fusion proteins, may be useful therapeutic proteins leading to efficient intracellular delivery of Hsp27 into hypoxic cells. In vivo efficacy of PTD-Hsp27 fusion proteins need to be evaluated in hypoxic diseases.

## References

[CR1] Arthanari Y, Pluen A, Rajendran R, Aojula H, Demonacos C (2010). Delivery of therapeutic shRNA and siRNA by Tat fusion peptide targeting bcr–abl fusion gene in chronic myeloid leukemia cells. J Control Release.

[CR2] Carter PJ (2011). Introduction to current and future protein therapeutics: a protein engineering perspective. Exp Cell Res.

[CR3] Del Gaizo V, Payne RM (2003). A novel TAT-mitochondrial signal sequence fusion protein is processed, stays in mitochondria, and crosses the placenta. Mol Ther.

[CR4] Deshayes S, Morris M, Divita G, Heitz F (2005). Cell-penetrating peptides: tools for intracellular delivery of therapeutics. Cell Mol Life Sci.

[CR5] Duchardt F, Fotin-Mleczek M, Schwarz H, Fischer R, Brock R (2007). A comprehensive model for the cellular uptake of cationic cell-penetrating peptides. Traffic.

[CR6] Gupta B, Levchenko TS, Torchilin VP (2005). Intracellular delivery of large molecules and small particles by cell-penetrating proteins and peptides. Adv Drug Deliv Rev.

[CR7] Herbig ME, Weller K, Krauss U, Beck-Sickinger AG, Merkle HP, Zerbe O (2005). Membrane surface-associated helices promote lipid interactions and cellular uptake of human calcitonin-derived cell penetrating peptides. Biophys J.

[CR8] Kaplan IM, Wadia JS, Dowdy SF (2005). Cationic TAT peptide transduction domain enters cells by macropinocytosis. J Control Release.

[CR9] Kwon JH, Kim JB, Lee KH, Kang SM, Chung N, Jang Y, Chung JH (2007). Protective effect of heat shock protein 27 using protein transduction domain-mediated delivery on ischemia/reperfusion heart injury. Biochem Biophys Res Commun.

[CR10] Lim KS, Won YW, Park YS, Kim YH (2010). Preparation and functional analysis of recombinant protein transduction domain-metallothionein fusion proteins. Biochimie.

[CR11] Liu L, Yu R, Shi Y, Dai Y, Zeng Z, Guo X, Ji Q, Wang G, Zhong J (2014). Transduced protein transduction domain linked HSP27 protected LECs against UVB radiation-induced damage. Exp Eye Res.

[CR12] Mishra A, Lai GH, Schmidt NW, Sun VZ, Rodriguez AR, Tong R, Tang L, Cheng J, Deming TJ, Kamei DT, Wong GC (2011). Translocation of HIV TAT peptide and analogues induced by multiplexed membrane and cytoskeletal interactions. Proc Natl Acad Sci USA.

[CR13] Schmidt N, Mishra A, Lai GH, Wong GC (2010). Arginine-rich cell-penetrating peptides. FEBS Lett.

[CR14] Shokolenko IN, Alexeyev MF, Ledoux SP, Wilson GL (2005). TAT-mediated protein transduction and targeted delivery of fusion proteins into mitochondria of breast cancer cells. DNA Repair.

[CR15] Snyder EL, Dowdy SF (2004). Cell penetrating peptides in drug delivery. Pharm Res.

[CR16] Takeuchi T, Kosuge M, Tadokoro A, Sugiura Y, Nishi M, Kawata M, Sakai N, Matile S, Futaki S (2006). Direct and rapid cytosolic delivery using cell-penetrating peptides mediated by pyrenebutyrate. ACS Chem Biol.

[CR17] Tan CY, Ban H, Kim YH, Lee SK (2009). The heat shock protein 27 (Hsp27) operates predominantly by blocking the mitochondrial-independent/extrinsic pathway of cellular apoptosis. Mol Cells.

[CR18] Tan ML, Choong PF, Dass CR (2010). Recent developments in liposomes, microparticles and nanoparticles for protein and peptide drug delivery. Peptides.

[CR19] Van Den Berg A, Dowdy SF (2011). Protein transduction domain delivery of therapeutic macromolecules. Curr Opin Biotechnol.

[CR20] Wadia JS, Stan RV, Dowdy SF (2004). Transducible TAT-HA fusogenic peptide enhances escape of TAT-fusion proteins after lipid raft macropinocytosis. Nat Med.

[CR21] Walsh G (2010). Biopharmaceutical benchmarks 2010. Nat Biotechnol.

[CR22] Wang W (2005). Protein aggregation and its inhibition in biopharmaceutics. Int J Pharm.

[CR23] Watson RW, Redmond HP, Wang JH, Bouchier-Hayes D (1996). Mechanisms involved in sodium arsenite-induced apoptosis of human neutrophils. J Leukoc Biol.

[CR24] Won YW, Kim HA, Lee M, Kim YH (2010). Reducible poly (oligo-d-arginine) for enhanced gene expression in mouse lung by intratracheal injection. Mol Ther.

[CR25] Zhang X, Zhang X, Wang F (2012). Intracellular transduction and potential of Tat PTD and its analogs: from basic drug delivery mechanism to application. Expert Opin Drug Deliv.

[CR26] Ziegler A (2008). Thermodynamic studies and binding mechanisms of cell-penetrating peptides with lipids and glycosaminoglycans. Adv Drug Deliv Rev.

